# Multi-carbohydrase strategies improve the utilisation of wheat distiller’s dried grains with solubles in broilers

**DOI:** 10.1016/j.psj.2025.106176

**Published:** 2025-12-02

**Authors:** Eunjoo Kim, Nishchal K. Sharma, Anna Fickler, Leon Hall, Mingan Choct

**Affiliations:** a School of Environmental and Rural Science, University of New England, Armidale, NSW 2350, Australia; b Poultry Hub Australia, University of New England, Armidale, NSW 2350, Australia; c Poultry Research Foundation, The University of Sydney, Camden 2570 NSW, Australia; d BASF SE, 67056 Ludwigshafen, Germany

**Keywords:** Broiler, β-mannanase, Carbohydrase cocktail, Wheat distillers dried grains with solubles, Xylanase and β-glucanase preparation

## Abstract

Wheat distiller’s dried grains with solubles (**DDGS**) are known for non-starch polysaccharide (**NSP**)-rich characteristics, limiting their use in poultry diets. This study evaluated whether enzyme supplementation enables higher inclusion of wheat DDGS in broiler diets. A total of 896 Cobb 500 mixed-sex broilers were assigned to 8 treatments in a 2 × 4 factorial arrangement with two levels of wheat DDGS (moderate: 60-108 g/kg; high: 187–224 g/kg) and four enzyme addition (none, xylanase+β-glucanase [**XG**], double-dose XG [**XG 2 ×**] or XG+β-mannanase [**XG+M**]) from d 0-35. During the starter phase (d0-10), birds fed the high wheat DDGS diet presented greater weight gain than those fed the moderate diet (*P* = 0.003); however, over d0-35, weight gain was reduced with the high wheat DDGS diet (*P* = 0.001). A wheat DDGS × enzyme interaction was observed for overall FCR (*P* < 0.001), where XG+*M* led to the most pronounced improvement in birds fed the high wheat DDGS diet but not in those fed the moderate diet. As main effects, the high wheat DDGS diet increased ileal digesta viscosity at d 35 (*P* < 0.001). All enzyme supplementation reduced ileal digesta viscosity at d 21 (*P* = 0.001) and d 35 (*P* = 0.025), and litter moisture at d 35 (*P* < 0.001). Ileal starch digestibility was improved (*P* = 0.016) by XG 2 × and XG+*M* compared with non-supplemented birds. In birds fed the moderate wheat DDGS diet, soluble NSP degradability was enhanced by XG 2 × compared with non-supplemented birds, whereas in birds fed the high wheat DDGS diet, it was improved by XG+*M*, leading to a significant interaction (*P* = 0.040). At the main effect level, total caecal short-chain fatty acid concentrations were higher in birds fed the high wheat DDGS diet (*P* = 0.020) and were increased by XG 2 × (*P* = 0.011). In summary, high wheat DDGS inclusion impaired growth performance, whereas XG+*M* mediated the greatest improvement in feed efficiency through enhanced soluble NSP degradation.

## Introduction

Starch-rich grains, mainly maize and wheat, are two of the primary global feedstocks for bioethanol production. Grain-based ethanol production generates significant quantities of distillers dried grains with solubles (**DDGS**), a valuable by-product for animal feed. Globally, maize is the predominant grain source for DDGS, and maize-based DDGS has been extensively studied in poultry nutrition. In contrast, the Australian ethanol industry predominantly uses wheat as the primary grain feedstock, resulting in wheat DDGS as the major by-product. According to Biofuels Annual report by the USDA Forign Agricultural Service (2024), wheat ethanol production in Eastern Australia has yielded approximately 0.36 tonnes of DDGS per tonne of wheat processed under typical dry-milling conditions (90 % DM basis). Based on more recent estimates, approximately 369,000 tonnes of wheat have been used annually for ethanol production in Australia, which equates to at least 130,000 tonnes of wheat DDGS available as by-product. This highlights the increasing availability of wheat DDGS for inclusion in poultry diets.

Wheat DDGS can be included in broiler diets to partially substitute maize and soybean meal, which can be beneficial to alleviate reliance on conventional feed ingredients. In practice, however, formulating a diet with wheat DDGS requires supplemental lysine and methionine as their availability can be reduced due to heat damage during processing (Bandegan et al., 2009; Cromwell et al., 1993). This often limits the inclusion of wheat DDGS up to 10 % in grower-finisher broiler diets (Thacker and Widyaratne, 2007). Another shortcoming of wheat DDGS is highly variable energy values depending on processing method and residual starch content (Gamage et al., 2012). Whiting et al. (2017) evaluated the feeding values of six different batches of wheat DDGS from a single production plant in broilers and found high variation in the nitrogen-corrected apparent matabolisable energy (**AME**), ranging from 3075 to 3235 kcal/kg DM when included at 150 g/kg in broiler diets. This variation may also stem from variations in the parent wheat grain. In Choct et al. (2006), nine wheat samples varied considerably in AME (2750-3250 kcal/kg DM), which was strongly and negatively correlated with total non-starch polysaccharides (**NSP**; *r* = –0.97, *P* < 0.001). A similar trend was reported by González-Ortiz et al. (2016), who found significant varietal differences of wheats in AME and nitrogen corrected-AME and showed that xylanase supplementation minimised performance variation across eight different wheat varieties in broilers. This clearly indicates that arabinoxylans are a major driver of the varietal variation of wheat in energy, and strategies that target this fraction can help reduce such differences.

The gravity of the issue becomes more evident when starch extraction from wheat substantially concentrates soluble arabinoxylans, other fibre fractions and Klason lignin in the resulting DDGS. Pedersen et al. (2014) quantified NSP contents in 11 wheat DDGS samples and reported a mean total NSP level of 26 % (CV 9 %), of which 25 % was soluble. Among polysaccharide constituents, pentoses (arabinose and xylose) accounted for 55 % of the total NSP, confirming a high proportion of arabinoxylans. By comparison, wheat grain contains about 9.7 % total NSP, with 23 % of this fraction being soluble (Smeets et al., 2014). This comparison highlights the enrichment of arabinoxylans in wheat DDGS and substantiates a strong rationale for the application of carbohydrases in wheat DDGS containing broiler feed.

To specifically investigate the nutritional implications of wheat DDGS, the present study used a maize-based diet as the background formulation. This avoided potential confounding effects from wheat itself, as wheat arabinoxylans could otherwise mask the impact of wheat DDGS inclusion. The objective of this study was to investigate whether different carbohydrase uses could overcome the challenges of incorporating wheat DDGS in broiler diets up to 200 g/kg. Specifically, we evaluated xylanase and β-glucanase preparation (**XG**) at recommended and double doses to target concentrated soluble arabinoxylans or a combination of XG and β-mannanase (**M**) to counteract multiple fibre structures. By evaluating these enzyme strategies, this research aims to enhance nutrient utilisation, mitigate the negative impacts of NSP-rich wheat DDGS on broiler performance when diets include wheat DDGS at levels up to 200 g/kg. Expanding the use of wheat DDGS has the potential to reduce feed costs and alleviate reliance on imported soybean meal, thereby aligning with sustainability goals in chicken meat production.

## Materials and methods

### Ethics statement

All experimental designs and procedures were thoroughly reviewed and approved by the Animal Ethics Committee at the University of New England (Authority No: ARA23-054).

### Experimental design, birds and housing

A total of 896 mixed-sex day-old Cobb 500 broiler chicks (Baiada, Tamworth, NSW, Australia) were used in a 2 × 4 factorial arrangement with two wheat DDGS inclusion levels (moderate or high) and four enzyme supplementation strategies (none, XG, XG double dose, or XG+M). This generated eight treatments, each with eight replicate pens of 14 birds. Birds were housed in the Multi-pulpose Building at University of New England in floor pens (1.12 m² excluding feeder space) with wood shavings bedding to a depth of 7 cm. Each pen was equipped with two nipple drinkers and a hanging tube feeder to provide *ad libitum* access to feed and water. Ambient temperature and lighting programs followed Cobb management guidelines, with temperature gradually reduced from placement to 22°C by 21 days of age.

### Experimental diets

The composition of experimental diets is presented in Table 1. The nutrient profile of maize, soybean meal and wheat DDGS were estimated using near-infrared reflectance spectrometry (trinamiX Inc., Ludwigshafen, Germany) prior to feed formulation. Diets were fed in three phases: starter (d 0–10), grower (d 10–21), and finisher (d 21–35). All diets were formulated to be approximately 10 % lower in essential amino acids than the Cobb 500 nutrient specifications (Cobb-Vantress, 2022) to allow scope for potential improvements with exogenous enzyme supplementation. The XG contained endo-1,4-β-xylanase activity of 5,600 TXU/g and endo-1,4-β-glucanase activity of 2,500 TGU/g (Natugrain® TS, BASF SE, Germany) and was included at 100 g/tonne of feed. The XG double dose treatment contained the same enzyme activities at an inclusion rate of 200 g/tonne. The M product supplied 8,000 TMU/g of endo-1,4-β-mannanase (Natupulse® TS, BASF SE, Germany) and was included at 100 g/tonne. Titanium dioxide was used as an indigestible marker at 5 g/kg. All diets contained phytase at 1,000 FTU/kg of feed (Natuphos® E, BASF SE, Germany), and matrix values for apparent metabolisable energy, essential amino acids, calcium, available phosphorus and sodium were applied according to manufacturer’s specification. Enzymes were added at the expense of sand and thoroughly mixed into the basal diets before pelleting. All diets were cold-pelleted at 65°C, and starter diets were subsequently crumbled.

**Table 1:** Composition of basal diets.

	Starter (d 0 - 10)		Grower (d 10 - 21)			Finisher (d 21 - 35)	
(g/kg as fed)	Moderate	High	Moderate	High		Moderate	High
Maize	622	589	614	571		647	583
Soybean meal 47 %	276	257	235	196		206	152
Wheat DDGS^1^	60	110	108	187		108	224
Canola oil	1.71	4.04	7.23	8.93		5.04	7.68
Limestone	9.99	9.47	9.46	10.56		9.13	10.72
Dical Phos 18P/21Ca	12.09	12.89	5.70	3.66		3.85	0.86
Sodium chloride	1.79	1.52	1.14	0.49		2.00	1.56
Na Bicarbonate	2.54	2.31	2.31	2.35		1.10	0.43
Sand	0.20	0.20	0.20	0.20		0.20	0.20
Titanium dioxide	-	-	5.00	5.00		5.00	5.00
Vitamin premix^2^	0.80	0.80	0.80	0.80		0.80	0.80
Mineral premix^3^	1.00	1.00	1.00	1.00		1.00	1.00
Choline Cl 60 %	0.51	0.67	0.55	0.84		0.69	1.09
L-lysine HCl	3.44	3.87	3.42	4.35		3.15	4.44
DL-methionine	3.33	3.28	2.95	2.94		2.60	2.58
L-threonine	1.56	1.62	1.22	1.41		1.99	2.24
L-Valine	0.95	0.91	0.57	0.63		0.41	0.45
L-Arginine HCl	1.43	1.81	1.38	2.23		1.32	2.49
Phytase^4^	0.10	0.10	0.10	0.10		0.10	0.10
Calculated values (g/kg)							
ME (kcal/kg)	2,930	2,930	2,980	2,980		3,000	3,000
Crude Protein	203	205	194	195		183	185
Dig Arg	12.24	12.24	11.25	11.25		10.40	10.40
Dig Lys	11.34	11.34	10.44	10.44		9.54	9.54
Dig Met	5.98	5.93	5.49	5.44		5.03	4.95
Dig Met+Cys	8.46	8.46	7.92	7.92		7.34	7.34
Dig Trp	2.07	2.05	1.93	1.85		1.78	1.68
Dig Leu	14.43	14.30	13.73	13.35		13.03	12.53
Dig Ile	7.29	7.29	6.90	6.77		6.42	6.26
Dig Thr	7.74	7.74	7.02	7.02		7.38	7.38
Dig Val	8.64	8.64	7.92	7.92		7.29	7.29
Calcium	9.60	9.60	8.00	8.00		7.40	7.40
Av. Phosphorous	5.00	5.40	4.00	4.00		3.60	3.60
Analysed values (g/kg as-is)							
Dry matter (%)	87.8	88.2	87.8	88.4		88.4	88.6
Crude protein	190	198	199	202		188	183
Starch	398	353	376	351		379	356
Total NSP^5^	73	79	83	94		78	94
Soluble	6.3	7.7	7.2	10.3		7.1	11.1
Insoluble	66	71	75	84		71	82
Free oligosaccharides	34	36	36	36		35	33

1 Wheat distiller’s dried grains with solubles.

2 Vitamin premix per kg diet (UNE VM, Rabar Pty Ltd): vitamin A, 12 MIU; vitamin D, 5 MIU; vitamin E, 75 mg; itamin K, 3 mg; nicotinic acid, 55 mg; pantothenic acid, 13 mg; folic acid, 2 mg; riboflavin, 8 mg; cyanocobalamin, 0.016 mg; biotin, 0.25 mg; pyridoxine, 5 mg; thiamine, 3 mg; antioxidant, 50 mg.

3 Mineral premix per kg diet (UNE TM, Rabar Pty Ltd): Cu, 16 mg as copper sulfate; Mn, 60 mg as manganese sulfate; Mn, 60 mg as manganous oxide; I, 0.125 mg as potassium iodide; Se, 0.3 mg; Fe, 40 mg, as iron sulfate; Zn, 50 mg as zinc oxide; Zn, 50 mg as zinc sulfate.

4 Natuphos E 10000 G (BASF SE, Germany); Matrix values for apparent metabolizable energy, essential amino acids, calcium, available phosphorus and sodium were applied.

5 NSP=Non-starch polysaccharides.

### Sample and data collection

On d 21 and 35, four birds per pen were selected and euthanised by electrical stunning followed by cervical dislocation for sample collection. The ileal and jejunal digesta samples were collected by gently expressing into containers and pooled by pen. The ileum was defined as the section from Meckel’s diverticulum to the ileocaecal junction, and the jejunum was as the section from the end of the duodenal loop to Meckel’s diverticulum. At d 35, caecal contents were collected and pooled per pen. All samples were stored at −20°C until analysis.

The jejunal and ileal digesta samples were homogenised for viscosity determination. Approximately 2 g of fresh digesta was transferred into 2 mL Eppendorf tubes and centrifuged at 16,000 × *g* for 10 min at room temperature. Viscosity of 0.5 mL supernatant was measured using a Brookfield DV3T Rheometer (Brookfield Ametek, Middleboro, MA, USA) fitted with a CPA-40Z spindle at 25°C, and results were expressed in centipoise (**cP**).

Digesta were then freeze-dried, ground to pass through a 0.5 mm sieve, and analysed alongside feed samples for nutrient digestibility calculation. Dry matter content was determined by oven-drying at 105°C to constant weight. Total starch was measured using a commercial assay kit (Megazyme, Bray, Ireland) with spectrophotometric detection. Soluble and insoluble NSP were quantified as alditol acetates by gas chromatography following Englyst et al. (1994) and Theander et al. (1995), using an Agilent 8890 GC with an Agilent 7693A Autosampler (Agilent Technologies, Palo Alto, CA, USA). A conversion factor of 0.90 was applied to convert monosaccharide values to polysaccharide values (Bach Knudsen, 1997). Titanium dioxide (**TiO2**) concentration was determined colorimetrically as described by Short et al. (1996). Spectrophotometric measurements for starch and titanium dioxide were performed using a Cary 50 Bio UV-Visible spectrophotometer (Varian Inc., Palo Alto, CA, USA) at 510 nm for starch and 410 nm for titanium dioxide. The feed enzyme activities were analysed using commercial kits according to manufacturer’s instructions (K-XYLS for xylanase, K-MBGL for β-glucanase and T-MNZ-200T for mannanase, Megazyme, Bray, Ireland)

Caecal contents were analysed for short-chain fatty acids (**SCFA**) following protocols adapted from Gu et al. (2021), Richardson et al. (1989) and Zhang et al. (2019). Approximately 450–600 mg of thawed samples were mixed with 1 mL of internal standard solution (0.01 M 2-ethyl butyric acid and 0.05 M succinic acid-2,2,3,3-d4 in 0.2 M NaOH), vortexed, and centrifuged at 12,000 g for 5 min. From the supernatant, 100 µL was acidified with 50 µL of 1 M HCl, extracted with 1.5 mL of diethyl ether, and dried over anhydrous sodium sulphate. A 50 µL aliquot of the ether phase was then mixed with 400 µL acetonitrile, 10 µL pyridine, and 25 µL MTBSTFA (1 % t-BDMCS), sealed, and derivatised at 80°C for 30 min. Derivatised samples were analysed by GC-MS (Agilent 7890A-5975C). SCFA peaks were identified and quantified using single-ion chromatograms of selected ions (m/z 103 for formic acid, 117 for acetic acid, 131 for propionic acid, 145 for butyric acid, 158 for valeric acid, 261 for lactic acid, 289 for succinic acid, 173 for 2-ethyl butyric acid, and 293 for succinic acid-d_4_). Results were expressed as µmol/g fresh digesta. All analyses were conducted in duplicate.

The following equation was used for apparent ileal nutrient digestibility or NSP degradability: Digestibility or degradability (%) = 1 − [(TiO_2_ in diet/TiO_2_ in digesta) × (nutrient in digesta/nutrient in diet)] × 100.

### Statistical analysis

All data were tested for normality prior to analysis. A two-way ANOVA was performed using IBM SPSS Statistics (Version 29, IBM Corp., Armonk, NY, USA), with pen as the experimental unit. The 2 × 4 factorial arrangement consisted of eight treatment combinations, each with 8 replicate pens. Means were separated using Tukey’s HSD test at *P* < 0.05, and trends were considered where P-values ranged from 0.05 to 0.10. Data that did not meet the assumptions of normality were analysed using the non-parametric Kruskal-Wallis test. The overall male-to-female ratio across all pens was 51:49, and the ratio of each pen did not have a significant effect on any variables tested.

## Results

Overall mortality over 35 days was 5.4 % and was not affected by wheat DDGS level, enzyme supplementation or their interaction.

### Experimental diets

The high DDGS diets contained slightly more crude protein (1-4 g/kg higher) but less starch (up to 27 g/kg lower) than the moderate DDGS diets. Soluble NSP concentrations were consistently higher in the high DDGS diets, by about 0.8-1.5 g/kg in the starter and grower phases and markedly higher (by 5.7 g/kg) in the finisher phase. Similarly, total NSP content was 2 g/kg higher in the grower and 16 g/kg higher in the finisher diets compared to their moderate DDGS counterparts. Insoluble NSP also increased in the high DDGS finisher diet (71.8 vs 61.7 g/kg). Enzyme activities of the experimental diets were measured (Table 2), and all were determined to be within the expected range.

**Table 2:** Analysed enzyme activities of experimental diets.

		Xylanase (560 XTU/kg)			Glucanase (250 XGU/kg)			Mannanase (800 TMU/kg)		
Wheat DDGS	Enzyme^2^	Starter	Grower	Finisher	Starter	Grower	Finisher	Starter	Grower	Finisher
Moderate	None	15	15	21	10	15	18	16	15	18
	XG	302	322	282	158	204	175	10	8	10
	XG 2 ×	616	608	582	332	333	378	17	15	24
	XG+*M*	329	283	292	168	166	152	916	1116	977
High	None	18	21	39	14	14	19	14	17	16
	XG	294	302	361	136	159	159	13	16	19
	XG 2 ×	633	617	679	303	320	326	6	7	16
	XG+*M*	356	371	358	122	126	162	895	913	904

### Growth performance

The influence of carbohydrase supplementation in the wheat DDGS-containing diets on the growth performance of broilers is summarised in Table 3. During the starter (d 0-10) phase, the high wheat DDGS diet led to increased BW gain (*P* = 0.003) compared with the moderate wheat DDGS diet. Enzyme supplementation tended to improve BW gain (*P* = 0.056), with XG and XG double-dose showing highest gains than the non-supplemented control. Neither feed intake nor FCR was affected by wheat DDGS level or enzyme supplementation, and no interactions were observed.

**Table 3:** Influence of carbohydrase supplementation on the performance of broilers fed the diet containing wheat distiller’s dried grains with solubles^1^.

		Weight gain (g/bird)				Feed intake (g/bird)				Feed conversion ratio^3^ (g/g)			
Wheat DDGS	Enzyme^2^	d 0-10	d 10-21	d 21-35	d 0-35	d 0-10	d 10-21	d 21-35	d 0-35	d 0-10	d 10-21	d 21-35	d 0-35
Moderate	None	258	783	1,421	2,463	309	975	2,177	3,461	1.196	1.246^bc^	1.534^c^	1.406^c^
	XG	268	785	1,429	2,483	313	972	2,168	3,453	1.168	1.238^c^	1.518^c^	1.390^c^
	XG 2 ×	266	802	1,452	2,519	310	988	2,184	3,482	1.165	1.234^c^	1.504^c^	1.381^c^
	XG+*M*	264	783	1,506	2,553	302	970	2,276	3,548	1.147	1.238^c^	1.516^c^	1.391^c^
High	None	266	761	1,170	2,198	307	1018	2,261	3,586	1.151	1.337^a^	1.933^a^	1.631^a^
	XG	276	765	1,355	2,400	315	1004	2,367	3,679	1.143	1.312^ab^	1.752^b^	1.535^b^
	XG 2 ×	278	763	1,362	2,403	326	973	2,195	3,493	1.174	1.275^abc^	1.612^bc^	1.455^bc^
	XG+*M*	272	775	1,434	2,481	305	956	2,309	3,570	1.120	1.235^c^	1.613^bc^	1.439^c^
SEM^4^		1.5	4.1	20.9	22.5	2.1	5.6	28.8	30.6	0.0070	0.0070	0.0207	0.0121
Main effects													
wDDGS level	Moderate	264^b^	788^a^	1,452^a^	2,505^a^	308	976	2,201	3,486	1.169	1.239	1.518	1.392
	High	273^a^	766^b^	1,330^b^	2,370^b^	313	988	2,283	3,582	1.147	1.290	1.728	1.515
Enzyme	None	262	772	1,296^b^	2,330^b^	308	997	2,219	3,523	1.174	1.292	1.733	1.519
	XG	272	775	1,392^ab^	2,441^ab^	314	988	2,267	3,566	1.156	1.275	1.635	1.463
	XG 2 ×	272	782	1,407^ab^	2,461^ab^	318	981	2,189	3,488	1.169	1.254	1.558	1.418
	XG+*M*	268	779	1,470^a^	2,517^a^	304	963	2,292	3,559	1.134	1.236	1.565	1.415
P-value													
Wheat DDGS		0.003	0.007	0.002	0.001	0.243	0.293	0.170	0.131	0.119	<0.001	<0.001	<0.001
Enzyme		0.056	0.805	0.013	0.012	0.066	0.168	0.587	0.800	0.177	0.005	<0.001	<0.001
Interaction		0.948	0.596	0.258	0.293	0.418	0.124	0.693	0.600	0.585	0.020	<0.001	<0.001

a-c. Values in a column with no common superscripts differ significantly (*P* < 0.05).

¹Mean values are based on 8 replicates per treatment, each pen containing 14 birds until day 21 and 10 birds thereafter.

2 None=no enzyme; XG=xylanase and beta-glucanase; XG 2 × = XG double-dosing; XG+*M*=XG plus mannanase.

3 Feed conversation ratio corrected for mortality.

4 SEM=standard error of the mean.

During the grower (d 10-21) phase, BW gain was reduced (*P* = 0.007) in birds fed the high wheat DDGS diet compared with the moderate wheat DDGS diet. Feed intake was not influenced by wheat DDGS level or enzyme supplementation. A wheat DDGS × enzyme interaction (*P* = 0.020) indicated that FCR improvements by enzyme supplementation were greater with the high wheat DDGS diet, where XG+*M* markedly improved FCR compared with the non-supplemented counterpart by 10 points.

During the finisher (d 21-35) phase, BW gain was lower (*P* = 0.002) in birds fed the high wheat DDGS diet compared with those fed the moderate wheat DDGS diet. Enzyme supplementation improved BW gain (*P* = 0.013), with XG+M supporting the highest gains. A wheat DDGS × enzyme interaction (*P* < 0.001) for FCR showed that the improvement occurred only in birds fed the high wheat DDGS diet, with XG+M and XG double dose producing a more pronounced improvement than XG.

Overall (d 0-35), high wheat DDGS inclusion reduced (*P* = 0.001) BW gain by 5.4 % compared with moderate wheat DDGS, without affecting feed intake. Enzyme supplementation improved BW gain (*P* = 0.012), with the combination of XG and M resulting in the highest gain. A wheat DDGS × Enzyme interaction (*P* < 0.001) for FCR showed that all enzyme treatments significantly improved FCR only in birds fed the high wheat DDGS diet.

### Digesta viscosity and litter moisture

The effects of carbohydrase supplementation and wheat DDGS inclusion level on digesta viscosity and litter moisture are presented in Table 4. At d 21, enzyme supplementation reduced (*P* = 0.001) ileal digesta viscosity compared with non-supplemented birds. At d 35, ileal digesta viscosity was higher (*P* < 0.001) in birds fed the high wheat DDGS diet compared with those fed the moderate wheat DDGS diet. Enzyme supplementation reduced ileal digesta viscosity (*P* = 0.025), with greater reductions observed for XG and XG double dose. Jejunal digesta viscosity was not altered by wheat DDGS inclusion level or supplemental enzymes.

**Table 4:** Effects of supplemental carbohydrases on small intestinal digesta viscosity (cP) and litter moisture (%) in broilers fed the diet containing wheat distiller’s dried grains with solubles^1^.

		d 21		d 35		
Wheat DDGS	Enzyme^2^	Jejunum	Ileum	Jejunum	Ileum	Litter moisture
Moderate	None	2.41	3.25	2.06	2.64	46.1
	XG	2.34	2.76	1.89	2.31	37.9
	XG 2 ×	2.24	2.85	2.15	2.48	41.9
	XG+M	2.42	3.02	2.00	2.43	38.8
High	None	2.48	3.59	2.03	3.09	46.1
	XG	2.18	3.10	2.19	2.71	41.9
	XG 2 ×	2.33	2.86	2.08	2.58	41.4
	XG+M	2.45	2.95	2.05	2.77	37.9
SEM^3^		0.034	0.057	0.042	0.051	0.65
Main effects						
Wheat DDGS	Moderate	2.35	2.97	2.03	2.46^b^	41.2
	High	2.36	3.13	2.08	2.79^a^	41.8
Enzyme	None	2.44	3.42^a^	2.04	2.87^a^	46.1^a^
	XG	2.26	2.93^b^	2.04	2.51^b^	39.9^bc^
	XG 2 ×	2.29	2.85^b^	2.11	2.53^b^	41.6^b^
	XG+M	2.44	2.99^b^	2.02	2.60^ab^	38.4^c^
P-value						
Wheat DDGS		0.911	0.133	0.507	<0.001	0.405
Enzyme		0.108	0.001	0.886	0.025	<0.001
Interaction		0.549	0.345	0.432	0.523	0.124

a,b. Values in a column with no common superscripts differ significantly (*P* < 0.05).

1 Mean values are based on 8 replicates per treatment with digesta pooled from 4 birds per replicate.

2 None=no enzyme; XG=xylanase and beta-glucanase; XG 2 × = XG double-dosing; XG+*M*=XG plus mannanase.

3 SEM=standard error of the mean.

Wheat DDGS inclusion level did not influence litter moisture, but enzyme supplementation reduced the content (*P* < 0.001). The lowest litter moisture was observed in birds supplemented with XG+M, followed by XG and then XG double dose. No wheat DDGS × Enzyme interactions were found for any of these measures.

### Apparent ileal nutrient digestibility and NSP degradability

The effects of carbohydrase supplementation and wheat DDGS inclusion level on apparent ileal nutrient digestibility and NSP degradability at day 35 are presented in Table 5. Enzyme supplementation improved ileal starch digestibility (*P* = 0.016), with XG double dose and XG+*M* leading to greater improvements. A wheat DDGS × enzyme interaction (*P* = 0.040) was detected for soluble NSP degradability. In the high wheat DDGS diet, XG+M markedly improved soluble NSP degradability compared with the non-supplemented control, whereas in the moderate wheat DDGS diet, the greatest improvement was observed with XG double dose. Insoluble NSP degradability was greater (*P* < 0.001) in birds fed the high wheat DDGS diet compared with those fed the moderate wheat DDGS diet.

**Table 5:** Effects of carbohydrases on apparent ileal nutrient digestibility (%) in 35 d-old broilers fed the diet containing wheat distiller’s dried grains with solubles^1^.

Wheat DDGS	Enzyme^2^	Starch	N	Soluble NSP^3^	Insoluble NSP	Free oligosaccharides
Moderate	None	95.6	68.6	−27.9^c^	−10.6	42.4
	XG	96.0	70.2	−6.2^abc^	−4.1	36.0
	XG 2 ×	97.2	72.5	4.9^ab^	1.4	39.5
	XG+M	97.2	68.5	−10.3^bc^	−3.5	32.6
High	None	95.2	68.1	−10.2^bc^	11.1	34.2
	XG	97.3	72.2	−2.5^ab^	19.9	34.8
	XG 2 ×	97.3	72.2	4.2^ab^	19.6	44.5
	XG+M	97.5	72.6	16.5^a^	18.8	41.9
SEM^4^		0.24	1.58	2.33	2.27	1.39
Main effects						
Wheat DDGS	Moderate	96.5	70.0	−9.9	−4.2^b^	37.6
	High	96.8	71.3	2.0	17.3^a^	38.9
Enzyme	None	95.4^b^	68.3	−19.1	0.3	38.3
	XG	96.6^ab^	71.2	−4.3	7.9	35.4
	XG 2 ×	97.3^a^	72.4	4.6	10.5	42.0
	XG+M	97.3^a^	70.5	3.1	7.7	37.3
P-value						
Wheat DDGS		0.514	0.242	0.002	<0.001	0.647
Enzyme		0.016	0.083	<0.001	0.243	0.381
Interaction		0.659	0.440	0.040	0.956	0.129

a-c. Values in a column with no common superscripts differ significantly (*P* < 0.05).

1 Mean values are based on 8 replicates per treatment with digesta pooled from 4 birds per replicate.

2 None=no enzyme; XG=xylanase and beta-glucanase; XG 2 × = XG double-dosing; XG+*M*=XG plus mannanase.

3 NSP=non-starch polysaccharides.

4 SEM=standard error of the mean.

### Caecal short chain fatty acid levels

The effects of carbohydrase supplementation and wheat DDGS inclusion level on caecal SCFA concentrations at d 35 are shown in Table 6. Birds fed the high wheat DDGS diet had higher (*P* = 0.002) acetic acid concentrations compared to those fed the moderate wheat DDGS diet. High wheat DDGS also resulted in lower isobutyric acid (*P* = 0.003) and isovaleric acid (*P* < 0.001) concentrations compared with the moderate wheat DDGS diet.

**Table 6:** Influence of carbohydrase supplementation on caecal short-chain fatty acids concentrations (µmol/g fresh caecal content) in 35 d-old broilers fed the diet with wheat distiller’s dried grains with solubles^1^^,^^2^.

Wheat DDGS	Enzyme	Acetic	Propionic	Butyric	Valeric	Isobutyric	Isovaleric	Succinic	Total SCFA^3^	BSCFA^4^:SCFA	Lactic
Moderate	None	72.0	6.7	19.9	1.6	0.75	0.43	1.1	101.5	0.012	2.1
	XG	74.7	6.8	20.4	1.7	0.76	0.40	1.3	104.8	0.011	2.3
	XG 2 ×	81.0	7.3	20.8	1.7	0.71	0.38	1.4	111.9	0.010	2.9
	XG+M	78.8	6.9	20.2	1.7	0.75	0.40	1.7	108.7	0.011	2.5
High	None	76.7	7.0	18.3	1.5	0.72	0.36	1.3	104.7	0.011	2.5
	XG	82.2	7.3	20.3	1.6	0.64	0.35	1.1	112.3	0.009	3.3
	XG 2 ×	87.5	7.1	23.2	1.7	0.60	0.31	2.4	120.4	0.008	3.0
	XG+M	83.1	7.2	22.7	1.6	0.58	0.29	1.8	115.5	0.008	3.0
SEM		1.01	0.16	0.48	0.03	0.018	0.012	0.12	1.49	0.0008	0.12
Main effects											
Wheat DDGS	Moderate	76.6^b^	6.9	20.3	1.7	0.74^a^	0.40^a^	1.4	106.7^b^	0.011^a^	2.4^b^
	High	82.4^a^	7.2	21.1	1.6	0.64^b^	0.32^b^	1.6	113.2^a^	0.009^b^	3.0^a^
Enzyme	None	74.4^b^	6.9	19.1	1.6	0.74	0.39	1.2	103.1^b^	0.011^a^	2.3
	XG	78.4^ab^	7.0	20.4	1.7	0.70	0.37	1.2	108.6^ab^	0.010^ab^	2.8
	XG 2 ×	84.2^a^	7.2	22.0	1.7	0.65	0.34	1.9	116.2^a^	0.009^b^	3.0
	XG+M	80.9^ab^	7.1	21.4	1.7	0.67	0.34	1.8	112.1^ab^	0.009^b^	2.7
P-value											
Wheat DDGS		0.002	0.492	0.415	0.289	0.003	<0.001	0.228	0.020	<0.001	0.022
Enzyme		0.002	0.927	0.160	0.758	0.326	0.269	0.072	0.011	0.025	0.219
Interaction		0.905	0.945	0.367	0.980	0.513	0.868	0.327	0.912	0.627	0.536

a,b. Values in a column with no common superscripts differ significantly (*P* < 0.05).

5. SEM=standard error of the mean.

1 Mean values are based on 8 replicates per treatment with caecal contents pooled from 4 birds per replicate.

2 None=no enzyme; XG=xylanase and beta-glucanase; XG 2 × = XG double-dosing; XG+*M*=XG plus mannanase.

3 Total SCFA = acetic acid + propionic acid + butyric acid + valeric acid + branched short-chain fatty acids.

4 BSCFA:SCFA=Total branched short-chain fatty acids to total short-chain fatty acids.

Enzyme supplementation increased (*P* = 0.002) acetic acid concentration, with XG double dose presenting the highest level. Total SCFA concentration was greater in birds fed a high wheat DDGS diet compared to a moderate wheat DDGS diet (*P* = 0.020), and in enzyme-supplemented birds compared to non-supplemented birds (*P* = 0.011), with XG double dose again showing the highest value, followed by XG+M. Lactic acid concentration was higher (*P* = 0.022) in birds fed the high wheat DDGS diet compared to those fed the moderate wheat DDGS diet. The branched SCFA (**BSCFA**) to total SCFA ratio was lower (*P* < 0.001) in birds fed the high wheat DDGS diet compared to those fed the moderate diet. XG 2 × and XG+M supplementation notably lowered (*P* = 0.025) the BSCFA:SCFA ratio when compared to non-supplemented control group.

## Discussion

It was anticipated that wheat DDGS would compromise growth performance, and the detrimental effects were more pronounced when the inclusion rate was high. The high wheat DDGS diet contained greater amounts of both soluble and insoluble NSP and less starch than the moderate wheat DDGS diet, which likely influenced nutrient availability for growth. Consequently, growth performance over 35 days was significantly impaired in the absence of supplemental carbohydrases when birds were fed the high wheat DDGS diet. The moderate inclusion of wheat DDGS, up to 108 g/kg in grower-finisher diets, comparably supported broilers to reach the Cobb500 straight-run objectives of 2479 g/bird weight gain and 1.44 g/g FCR at d 35 (Cobb-Vantress, 2022). This finding is encouraging, as it suggests that wheat DDGS can partially replace conventional raw materials without compromising performance, which may offer economic benefits depending on local grain and protein meal markets. However, higher inclusions of wheat DDGS up to 224 g/kg in grower-finisher diets reduced weight gain by 11 % and worsened FCR by 16 %, clearly indicating that strategies are required to counteract the negative impacts of wheat DDGS at higher levels.

It was surprising that during the starter phase, birds fed the high wheat DDGS diet showed greater weight gain than those fed the moderate diet, which was not the true for the later growth phases. This early response was likely due to greater digesta retention in the foregut, as fibre-rich characteristics of the high wheat DDGS diet may have increased gut fill rather than true tissue accretion. The more NSP content in the high wheat DDGS inclusion, particularly insoluble fractions, can swell and hold large amounts of water, possibly increasing digesta bulk and contributing to apparent BW (Hetland et al., 2004). Given that the birds were small during this stage, the relative contribution of digesta mass to BW would have been proportionally greater, thus appearing as a significant increase in weight gain. Indeed, this apparent early increase in weight gain was only transient, likely diminishing as the birds matured, gut capacity became proportionally smaller relative to body size, and the negative impacts of excessive NSP associated with wheat DDGS became more pronounced.

An explanation for the notable inferiority of the high wheat DDGS diets is not straightforward in the present study. Although the high DDGS diets created a more viscous ileal environment, apparent starch and protein digestibility were not influenced by wheat DDGS levels. It is reasonable to assume that both moderate and high inclusions penalised nutrient utilisation to some degree, as exogenous enzyme supplementation improved ileal starch digestibility regardless of inclusion level. The more plausible interpretation, however, is that inefficiencies in energy utilisation beyond apparent nutrient digestibility accounted for the compromised performance of birds fed the high wheat DDGS diet. NSP-rich by-products are known to stimulate endogenous losses and mucin secretion and to create a viscous intestinal environment (Ito et al., 2009; Kluth and Rodehutscord, 2009; Morel et al., 2005), all of which elevate the heat increment of feeding. Consequently, such ingredients often exhibit lower net energy efficiency compared with cereal grains in non-ruminant animals. To our knowledge, no study has directly evaluated the net energy value of wheat DDGS in poultry, but the gap between NE and AME when feeding fibre-rich by-products is not new (Adeola and Kong, 2014; Cerrate et al., 2019; Wu et al., 2019). Wheat DDGS also contains relatively high crude protein with a poor, unbalanced amino acid profile (Cozannet et al., 2010). Diets high in protein, particularly when fractions are poorly digestible, combined with substantial insoluble NSP and lignin, are likely to exacerbate endogenous secretions and epithelial protein turnover. These processes are metabolically demanding, consuming energy without contributing to growth, thereby substantially reducing the true energy value of wheat DDGS available for growth and maintenance in poultry.

Total SCFA concentrations in the caeca increased with higher wheat DDGS inclusion, showing that more undigested but fermentable substrates escaped small intestinal digestion and ended up being metabolised by the hindgut microbiota. Double dosing of XG led to a greater acetic acid and total SCFA production in the caeca, though this did not necessarily translate into the most pronounced improvement in growth performance. These results suggest that either the presence of more fibrous substrates or extensive NSP hydrolysis to fermentable short-chain oligosaccharides by exogenous enzymes can favour microbial fermentation. BSCFA are produced when branched chain amino acids from undigested protein are fermented in the hindgut, while SCFA are the resultants of microbial fermentation of fibres and complex carbohydrates (Liu et al., 2021). The BSCFA to SCFA ratio is therefore an indication of whether microbial fermentation is driven more by residual protein or by fermentable fibre. Wheat DDGS diet at the higher inclusion reduced the BSCFA:SCFA ratio compared with the moderate inclusion, suggesting that increasing dietary fibre level can shift towards fibre, rather than nitrogen fermentation. Supplementing double XG and the XG+M combination also promoted fibre fermentation, as shown by the lower BCFA to SCFA ratio, highlighting the presence of more fermentable short-chain oligosaccharides by NSP hydrolysis.

Increased SCFA production from microbial fibre fermentation can help maintain an acidic gut environment, which is less favourable for pathogenic bacteria, thereby reducing their colonization and infection risk (Lamas et al., 2019; Liu et al., 2021; Kim et al., 2022c). Acetate is a primary SCFA in the caeca of poultry and serves as an energy substrate for enterocytes and peripheral tissues, supporting overall metabolism (Zhang et al., 2023). These prebiotic benefits should not be overlooked; however, the fermentation-derived energy is captured much less efficiently than glucose absorbed in the small intestine (Jamroz et al., 2002). Elevated lactate concentrations in birds fed the high wheat DDGS diets also indicate intensified microbial fermentation of soluble carbohydrates. In mammalian models, microbiota-derived lactate has been shown to signal epithelial hyperproliferation (Okada et al., 2013). While direct evidence in poultry is limited, such impacts would be consistent with increased epithelial turnover and higher maintenance energy costs, contributing to the lower net energy efficiency of wheat DDGS. Taken together, these findings suggest that while enzyme-mediated fermentation effects might support gut health, the true net energy value of wheat DDGS deserves much closer evaluation.

As expected, total NSP concentrations were 21 % higher and soluble NSP 56 % higher in the high wheat DDGS diet compared with the moderate diet. These soluble fractions likely contributed to the elevated digesta viscosity and altered caecal fermentation patterns observed. Carbohydrase supplementation counteracted these effects, with XG and the XG+M combination improving ileal NSP degradability and lowering digesta viscosity, thereby alleviating antinutritive impact of NSP on growth performance. The negative NSP degradability observed in the present study is likely due to the accumulation of NSP in the small intestine. Poultry lack endogenous enzymes to hydrolyse NSP, so these carbohydrate fractions remain undegraded in the gastrointestinal tract and are not converted to absorbable forms (Kim et al., 2022b). Supplementing carbohydrases improved NSP degradability either numerically or statistically, making the values less negative. This was most evident with double XG dosing in the moderate wheat DDGS diet and with XG+M in the high DDGS diet, highlighting increasing enzyme dose or combining enzyme activities can be more effective under higher NSP loads. Apparent ileal starch digestibility was also enhanced when XG was supplied at double dose or in combination with M, regardless of DDGS inclusion level. This outcome can be attributed to reduced viscosity and the partial breakdown of cell wall barriers, which allowed greater access of endogenous enzymes to starch (Choct et al., 2006; Kim et al., 2022d). The magnitude of FCR improvement mediated by enzymes was more pronounced in birds fed the high wheat DDGS diet, whereas only numerical gains were observed under the moderate DDGS diet. This interaction indicates that enzyme efficacy is strongly substrate-dependent, with higher NSP concentrations, particularly soluble fractions, providing greater opportunity for enzymatic action (Kim et al., 2022a, 2022d). Given that arabinoxylans are the predominant fibre fraction in wheat DDGS (Pedersen et al., 2014), XG alone alleviated viscosity and improved FCR in birds fed the high wheat DDGS diet.

The pattern of improvement followed a clear progression (none < XG < XG 2 × < XG+M), showing that both enzyme dose and substrate range are critical when dietary NSP levels are substantially elevated. A single inclusion of XG at the commercially recommended level was sufficient to reduce viscosity and improve FCR, while doubling the dose further enhanced NSP degradation and efficiency. More notably, adding β-mannanase extended hydrolysis beyond arabinoxylans to include other fibre fractions, exerting broader hydrolytic effect that consequently restored FCR in the high wheat DDGS diet to a level comparable with the moderate wheat DDGS diet. These findings highlight that enzyme efficacy is not simply additive but depends on aligning enzyme dose and specificity with the fibre load and composition of the diet, an especially important consideration when formulating with fibre-rich by-products.

## Conclusion

This study demonstrates that wheat DDGS can be moderately included in broiler diets (up to 108 g/kg) without compromising growth performance. However, higher inclusions (224 g/kg) may reduce weight gain and worsen feed efficiency, likely due to elevated dietary NSP and imbalances in nutrient utilisation. Carbohydrase supplementation, particularly xylanase and β-glucanase at higher dose or in combination with β-mannanase, alleviated NSP-induced viscosity, improved ileal starch digestibility, and restored feed conversion efficiency in high wheat DDGS diets to levels comparable with moderate inclusion. These findings emphasise that enzyme efficacy is strongly substrate-dependent and that matching enzyme dose and activity spectrum to the fibre composition of diets is critical when formulating with fibre-rich by-products such as wheat DDGS. Hence, the true net energy value of wheat DDGS remains poorly defined, and future evaluation is warranted to more accurately determine its contribution to poultry diets and enable more practical and efficient use.

## CRediT authorship contribution statement

**Eunjoo Kim:** Writing – original draft, Methodology, Investigation, Formal analysis, Data curation, Conceptualization. **Nishchal K. Sharma:** Writing – review & editing, Data curation. **Anna Fickler:** Writing – review & editing, Resources, Conceptualization. **Leon Hall:** Writing – review & editing, Resources, Conceptualization. **Mingan Choct:** Writing – review & editing, Supervision, Funding acquisition, Conceptualization.

## Disclosures

The authors declare that this research was carried out without any commercial or financial relationships that might be seen as a potential conflict of interest.
